# A Survey of the Presence of Pharmaceutical Residues in Wastewaters. Evaluation of Their Removal using Conventional and Natural Treatment Procedures

**DOI:** 10.3390/molecules25071639

**Published:** 2020-04-02

**Authors:** R. Guedes-Alonso, S. Montesdeoca-Esponda, J. Pacheco-Juárez, Z. Sosa-Ferrera, J. J. Santana-Rodríguez

**Affiliations:** Instituto Universitario de Estudios Ambientales y Recursos Naturales (i-UNAT), Universidad de Las Palmas de Gran Canaria, 35017 Las Palmas de Gran Canaria, Spain; sarah.montesdeoca@ulpgc.es (S.M.-E.); javier.pacheco@ulpgc.es (J.P.-J.); zoraida.sosa@ulpgc.es (Z.S.-F.); josejuan.santana@ulpgc.es (J.J.S.-R.)

**Keywords:** wastewaters, pharmaceutical residues, constructed wetlands, conventional wastewater treatments, solid phase extraction, ultra-high performance liquid chromatography

## Abstract

To encourage the reutilization of treated wastewaters as an adaptation strategy to climate change it is necessary to demonstrate their quality. If this is ensured, reclaimed waters could be a valuable resource that produces very little environmental impact and risks to human health. However, wastewaters are one of the main sources of emerging pollutants that are discharged in the environment. For this, it is essential to assess the presence of these pollutants, especially pharmaceutical compounds, in treated wastewaters. Moreover, the different treatment processes must be evaluated in order to know if conventional and natural treatment technologies are efficient in the removal of these types of compounds. This is an important consideration if the treated wastewaters are used in agricultural activities. Owing to the complexity of wastewater matrixes and the low concentrations of pharmaceutical residues in these types of samples, it is necessary to use sensitive analytical methodologies. In this study, the presence of 11 pharmaceutical compounds were assessed in three different wastewater treatment plants (WWTPs) in Gran Canaria (Spain). Two of these WWTPs use conventional purification technologies and they are located in densely populated areas, while the other studied WWTP is based in constructed wetlands which purify the wastewaters of a rural area. The sampling was performed monthly for two years. A solid phase extraction (SPE) coupled to ultra-high performance liquid chromatography tandem mass spectrometry (UHPLC-MS/MS) method was applied for the analysis of the samples, and the 11 pharmaceuticals were detected in all the studied WWTPs. The concentrations were variable and ranged from ng·L^−1^ in some compounds like diclofenac or carbamazepine to µg·L^−1^ in common pharmaceutical compounds such as caffeine, naproxen or ibuprofen. In addition, removal efficiencies in both conventional and natural purification systems were evaluated. Similar removal efficiencies were obtained using different purifying treatments, especially for some pharmaceutical families as stimulants or anti-inflammatories. Other compounds like carbamazepine showed a recalcitrant behavior. Secondary treatments presented similar removal efficiencies in both conventional and natural wastewater treatment plants, but conventional treatments showed slightly higher elimination ratios. Regarding tertiary system, the treatment with highest removal efficiencies was reverse osmosis in comparison with microfiltration and electrodialysis reversal.

## 1. Introduction

The reuse of reclaimed waters for irrigation could represent an alternative to the scarcity of water in arid locations, in addition to saving fertilizers and avoiding the discharge of this water into the environment, which has important ecological impacts [1]. However, the reuse of reclaimed waters is controversial because of the possible presence of emerging contaminants and their entry into the food chain [2]. Wastewaters have been revealed as one of the main pathways of introduction of these types of compounds into the environment. Thus, the increasing demand of water and discovery of the harmful effects of the emerging pollutants over aquatic biota suggest that continuous monitoring of wastewaters is needed [3].

Among these types of pollutants, pharmaceutical compounds are of great concern, not only for legislators, but also the scientific community, due to the variety of physic-chemical properties and the many toxic effects of them. Moreover, this concern is also related to the large consumption of these compounds in modern society, which is estimated as several tons in the European Union alone [4].

The harmful effects of pharmaceuticals over biota are produced when these compounds reach aquatic compartments through wastewaters and organisms are exposed to them [5]. This exposure is due to purification technologies of wastewater treatment plants (WWTPs) which are not designed to eliminate emerging pollutants [6,7]. Moreover, current legislation, does not establish limits for the concentrations of pharmaceuticals in environmental waters. The elimination of pharmaceutical compounds from wastewaters is significantly complex due to the variety of these analytes, their properties, and the possible conjugated compounds formed during metabolization [8]. Nevertheless, in 2015 the European Commission created a watch list which “is a mechanism for obtaining high-quality Union-wide monitoring data on potential water pollutants for the purpose of determining the risk they pose and thus whether Environmental Quality Standards (EQS) should be set for them at EU level” [9]. In this watch list, one anti-inflammatory compound, diclofenac, was added because of the well-known harmful effects of this compound to the aquatic environment [9].

Many studies have focused on determining the occurrence of pharmaceutical compounds in environmental samples. In this sense, some compounds like caffeine and nicotine and their respective metabolites paraxanthine and cotinine, and some broadly used drugs like naproxen or acetaminophen are usually detected at higher concentrations (in the range of µg·L^−1^) than other pharmaceuticals. Other common pharmaceuticals like diclofenac, erythromycin or carbamazepine are usually in the range of ng·L^−1^ as previous research has indicated [10,11,12]. However, many studies evaluate the occurrence of pharmaceuticals during a short period of time; few studies examine these compounds in the long term to evaluate seasonal fluctuations and determine patterns from the presence of these compounds.

In light of the above, it is necessary to carry out monitoring studies of pharmaceuticals to establish not only if the recipient aquatic ecosystems could be contaminated by these kinds of residues, but also to evaluate the efficiency of wastewater treatment facilities in the removal of these compounds. The removal of pharmaceuticals is not always achieved in WWTPs and, as some authors have determined, sometimes this degradation is partial and, in most cases, inefficient [13,14]. For this, it is necessary to evaluate the efficiency of conventional and natural wastewater treatments in the removal of pharmaceutical residues. Conventional wastewater treatments are employed in medium and largely populated areas to purify urban wastewaters, while natural treatments use the purifying power of bacteria, soils, and plants to treat wastewaters. Natural treatments are appropriate for the treatment of domestic wastewaters of small or isolated areas because they require large land areas to be settled. Nonetheless, their advantages are greater than their disadvantages because natural wastewater treatment systems have a low impact on the landscape, use minimal energy and little to no chemicals, and they produce relatively lower amounts of residual solids [15]. In spite of their differences, both systems have shown their capabilities to degrade and remove emerging pollutants from wastewaters [16,17,18].

In this study, three WWTPs, two based on conventional treatment technologies and one based on natural purification processes, were surveyed for two years in order to determine the occurrence and removal of pharmaceutical residues in Gran Canaria island (Spain). Eleven different compounds (Table 1) belonging to different pharmaceutical families (anti-inflammatories, stimulants, lipid regulators, antihypertensives, anticonvulsants, and antibiotics) were determined in different purifying stages of the studied WWTPs in order to determine the efficiency of conventional and natural wastewater treatments in the removal of them. The choice of these 11 pharmaceuticals was influenced by a previous study that monitored the same WWTPs for six months and showed that these 11 pharmaceuticals were the most detected compounds from a group of 23 different drugs [19]. Due to the expected low concentrations of the compounds, solid phase extraction (SPE) was used as an extraction and preconcentration technique. Then, ultra-high performance liquid chromatography tandem mass spectrometry (UHPLC–MS/MS) was used as the detection and determination technique.

## 2. Materials and Methods

### 2.1. Materials

The target pharmaceuticals were purchased from Sigma-Aldrich (Madrid, Spain) and presented purities over 97%. Three internal standards (IS), atenolol-d7 (Toronto Research Chemical Inc, Toronto, Canada), ibuprofen-d3 (Sigma-Aldrich, Madrid, Spain), and sulfamethoxazole-d4 (Dr. Ehrenstorfer GmbH, Ausgburg, Germany) were used to minimize the matrix effect of the studied samples. 

Stock solutions were prepared at 1000 mg·L^−1^ by dissolving the compound in high-purity methanol. These solutions were stored in glass-stoppered bottles at –20 °C prior to use. Working solutions were prepared daily from a stock mixture of 10 mg·L^−1^ by diluting proper quantities of the stock mixture in LC–MS grade water.

The mobile phase of UHPLC–MS/MS system was prepared using LC–MS grade methanol and water, both purchased from Panreac (Barcelona, Spain) as well as the modifiers of the mobile phase. The Milli-Q water used in the wash step of SPE was obtained using a water purification system of Millipore (Bedford, MA, USA)

### 2.2. Sample Collection

Samples were taken in three different wastewater treatment plants from Gran Canaria island (Spain). Two use conventional treatment technologies (C-WWTP1 and C-WWTP2) and receive wastewater from highly-populated areas of the island, located in the northeast and the southeast, respectively. C-WWTP1 has a secondary treatment of activated sludge and a tertiary system based on microfiltration and reverse osmosis. This WWTP treats the water of a population equivalent to 134,000 inhabitants. In C-WWTP2 only the tertiary process based on ultra-filtration was evaluated in order to know the possible risk of the produced water. This WWTP treats the water of a population equivalent to 200,000 inhabitants. The third WWTP surveyed is based in natural treatments (N-WWTP) and is located in a rural zone of the island. It is based in two constructed wetlands (CWs), the first one is a vertical flow wetland and the second one is a planted sub-superficial horizontal flow wetland. This WWTP was designed to treat wastewater equivalent to 500 inhabitants but nowadays it treats a higher volume of wastewater with great results.

**Figure 1:**
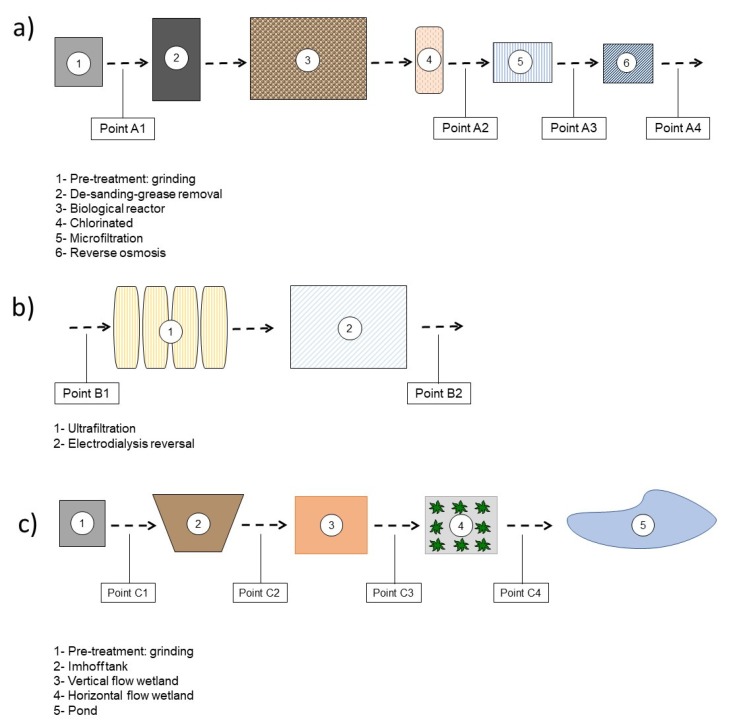
Layout of wastewater treatment plants (WWTPs) surveyed. (**a**) Conventional Wastewater Treatment Plant 1 (C-WWTP1); (**b**) Conventional Wastewater Treatment Plant 2 (C-WWTP2); (**c**) Natural Wastewater Treatment Plant (N-WWTP). Figure adapted from [19].

In C-WWTP1, the samples were taken in the influent of the plant (point A1), after the biological treatment (point A2), after the microfiltration process (point A3) and lastly, in the final effluent of the plant, after the reverse osmosis process (point A4). In C-WWTP2, the samples were taken before and after the electrodialysis reversal process (points B1 and B2). Finally, in N-WWTP, the samples were taken in the influent (point C1) and after each process of the treatment: Imhoff tank (point C2), vertical flow wetland (point C3), and horizontal sub-superficial flow wetland (point C4) (Figure 1).

The samples were taken monthly for two years from July 2017 to June 2019, at the same time slot and collected in rinsed amber bottles of 1 L. Samples were filtered using 0.65 µm polyvinylidene fluoride (PVDF) membrane filters from Merck Millipore (Cork, Ireland), and acidified to pH below 3 using hydrochloric acid and stored in the dark at 4 °C to inhibit microbial activity.

### 2.3. Analytical Methodology

To extract and preconcentrate the target pharmaceuticals in wastewater samples, a methodology based on SPE previously optimized [19] was used. Briefly, 250 mL of filtered wastewater at pH 7 were extracted using 500 mg Oasis HLB cartridges (Waters, Barcelona, Spain) in a Varian SPE manifold (Madrid, Spain). After the loading of the samples, the cartridges were washed with 5 mL of Milli-Q water and after that, the retained compounds were eluted with 5 mL of methanol. To achieve a great preconcentration factor, the solvent was evaporated under a gentle nitrogen steam and reconstituted with 1 mL of Milli-Q water with 100 µg·L^−1^ of internal standards. Before analysis the extracts were filtered using Chromafil Xtra PET-20/25 syringe filters with a pore size of 0.20 µm from Machery-Nagel (Düren, Germany).

After the extraction, ultra-high performance liquid chromatography tandem mass spectrometry (UHPLC–MS/MS) was used as a separation and detection technique. The separation of pharmaceuticals was performed using an ACQUITY UPLC BEH Waters C18 column (50 mm × 2.1 mm, 1.7 µm) from Waters Chromatography (Barcelona, Spain). The mobile phase used consisted of LC–MS grade water and methanol both with 0.5% acetic acid and the separation of target compounds was done in gradient mode (Table S1). The chromatographic system used was an AQCUITY UPLC system and consisted of a binary solvent manager pump, an autosampler, a column manager, and a triple quadrupole detector (TQD) controlled by Masslynx software, all of them from Waters Chromatography (Barcelona, Spain). The detection of the target compounds was carried out using electrospray ionization (ESI) in both positive and negative mode and the mass spectrometer parameters had a capillary voltage of 3.00 kV in positive mode and –2.50 kV in negative mode, source temperature of 120 °C and desolvation temperature of 450 °C. The conditions of fragmentation and the ions of the pharmaceuticals under study are presented in Table S2.

This analytical methodology presented great recoveries, between 52.9% and 123.6%, and appropriate detection limits, between 15.3 ng·L^−1^ and 13.3 µg·L^−1^. The method also showed great linearity, with correlation coefficients (r^2^) over 0.99 in all cases and good intra-day and inter-day repeatability, with relative standard deviation (RSD) values below 22% [19].

## 3. Results and Discussion

In this study, solid phase extraction (SPE) coupled to ultra-high-performance liquid chromatography tandem mass spectrometry (UHPLC-MS/MS) method was applied for analysis of water samples from three WWTPs. After the analysis, a statical study was performed in order to establish the median concentrations of the detected pharmaceuticals. The median concentration was used instead of the average concentration due to the dispersion of the concentrations detected during the two years of study. Moreover, the frequency of detection and the removal efficiency of each purification process were also calculated by dividing the number of samples with detected concentrations of a pharmaceutical by the total number of analyzed samples (positive results in Table 2).

### 3.1. Occurrence and Concentrations of Target Pharmaceuticals

#### 3.1.1. Conventional Treatment WWTPs

Conventional wastewater purification treatments are based on a primary treatment which is focused on the elimination of fats, sand, and coarse solids. After that, a secondary treatment, usually based on biological processes, is used to degrade organic matter from wastewater as well as to eliminate suspended solids and other organic pollutants. These processes could be aerobic or non-aerobic, and the most used biological process is activated sludge. Finally, if the wastewater will be re-used, tertiary processes must be performed in order to obtain a water with great quality. There are many tertiary treatment technologies and most used are microfiltration, nanofiltration or reverse osmosis [20,21,22,23].

**Figure 2:**
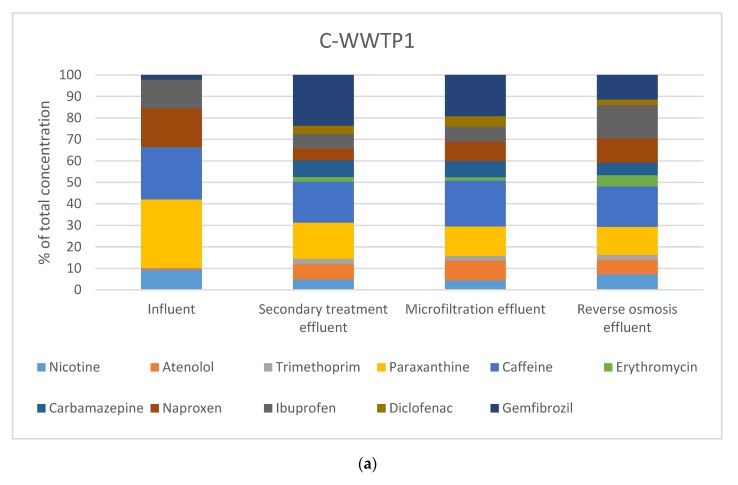

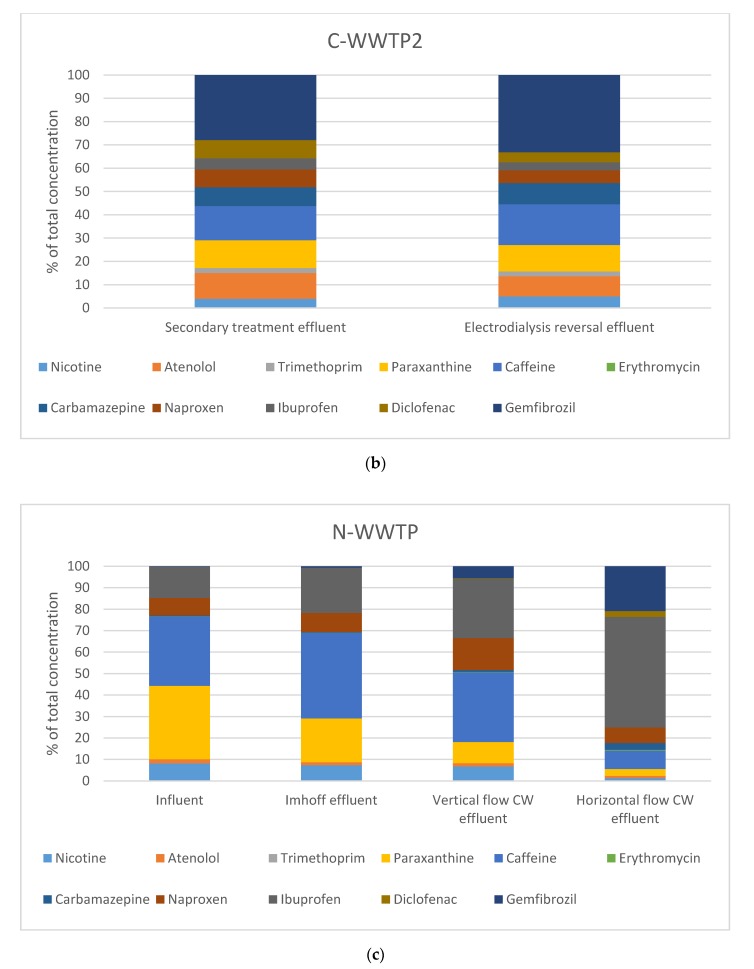
Distribution of pharmaceuticals from Table 1. (**a**) C-WWTP1, (**b**) C-WWTP2, (**c**) N-WWTP.

C-WWTP1 is based in activated sludge technology and as can be seen in Table 2, the stimulants under study (nicotine, caffeine, and paraxanthine) present the highest concentrations at influent samples, probably because these compounds are used in pharmaceutical formulations but also are excreted by smokers in the case of nicotine and after drinking beverages like coffee in the case of caffeine and its metabolite, paraxanthine. In fact, the median concentrations of these compounds reached values between 45.8 and 95.6 µg·L^−1^ during monitoring. This behavior was observed in other studies in which the highest concentrations of pharmaceuticals studied were obtained for these three stimulants as well [24,25,26,27]. Moreover, two anti-inflammatory drugs, naproxen and ibuprofen, also presented median concentrations in the range of µg·L^−1^ (between 19.9 and 27.3 µg·L^−1^) in influent samples and naproxen showed the highest concentration of all the monitored compounds (521.7 µg·L^−1^ in an influent sample). Anti-inflammatory compounds show a high rate of consumption in Spain [28] and the high concentrations of ibuprofen can be explained because this compound does not require a medical prescription as some authors have stated [29]. In addition, two pharmaceuticals related to cardiovascular problems and diseases namely, atenolol and gemfibrozil, presented low influent median concentrations of µg·L^−1^ (1.13 and 3.35 µg·L^−1^, respectively). The median concentrations of the rest of the target compounds were in the range of ng·L^−1^. As the purification process was performed, the median concentration of the compounds decreased. In fact, the sum of the median concentrations of the target compounds ranged from 150.9 µg·L^−1^ in the influent, to 0.90 µg·L^−1^ after the osmosis treatment, which is indicative of great elimination in the wastewater purification system. Figure 2a shows the changes in the distribution of pharmaceutical median concentrations in the different sampling points. In this sense, it can be observed that the contributed total concentration of stimulants decreased during the purification process, while the contribution of some compounds, like carbamazepine, increased; this is because the concentrations during the purification process remain stable. In fact, carbamazepine’s median concentration was similar in points A1 and A2 (0.146 and 0.052 µg·L^−1^, respectively), but the contribution to the total concentration changed from 0.1% to 5.7% after secondary and tertiary treatment. The composition of the final effluent obtained in the WWTP is a key factor, because it will be useful to predict the possible effects of effluents used in agriculture or discharged into the environment.

C-WWTP2 has an activated sludge treatment too, but in this WWTP the study was conducted in the tertiary process which is based on electrodialysis reversal. This technology has been demonstrated as an effective way to remove some emerging pollutants from drinking water [30] but studies in wastewaters are scarce and for this reason, this tertiary process was evaluated. In this wastewater treatment plant, the samples were taken after the secondary process (point B1) and after the electrodialysis technology (point B2). Before this treatment, an ultrafiltration process was also performed as pretreatment. Ten of the 11 pharmaceuticals under study were detected at concentrations that ranged from 0.055 to 0.723 µg·L^−1^ in point B1 and from 0.043 to 0.701 µg·L^−1^ in point B2. The highest concentrations were of gemfibrozil, caffeine, and paraxanthine, which ranged from 0.241 to 0.723 µg·L^−1^; this coincides with the results of C-WWTP1. This same behavior can be explained because both WWTPs treat the water of big urban areas with similar characteristics. In the same way, the lowest concentrations after secondary treatments (points A2 and B1) coincide in the two conventional WWTPs and correspond to erythromycin, nicotine, and trimethoprim. Figure 2b shows a similar distribution of pharmaceuticals before and after the purification process which means that this technology had a similar impact in the removal of the target compounds.

#### 3.1.2. Natural Treatment WWTP

An alternative to conventional WWTPs is natural WWTPs. From the different types of natural treatment technologies, constructed wetlands have revealed themselves as a great alternative to treat municipal wastewaters from small communities or isolated areas in both vertical flow and horizontal flow layouts [31,32]. In this system, the purification of wastewater is partially done in the vertical flow wetland and the horizontal flow wetland improves the water quality. The highest concentrations of target pharmaceuticals match with C-WWTP1, and were from caffeine, paraxanthine, nicotine, ibuprofen, and naproxen. All of these compounds presented median concentrations that ranged from 9.60 to 40.05 µg·L^−1^, while the rest of the target compounds were in the range of ng·L^−1^. The highest concentrations detected in the influent samples of this WWTP were from naproxen (320.07 µg·L^−1^) and caffeine (126.40 µg·L^−1^). As in the previous WWTPs, in this WWTP the concentrations decreased as the purification process was conducted. This can be observed in the total median concentrations of the WWTP that were 117.02 µg·L^−1^ in the influent (point C1), 104.14 µg·L^−1^ after Imhoff treatment (point C2), and 51.61 and 19.06 µg·L^−1^ after vertical flow (point C3) and horizontal flow (point C4) wetlands, respectively. Figure 2c shows the distribution of pharmaceuticals in the sampling points and it can be observed that the distribution in the influent of this WWTP is similar to C-WWTP1. In the influents of both WWTPs, the majority of compounds found were stimulants and anti-inflammatories. Nevertheless, the pharmaceutical profiles were different in the rest of the sampling points due to the removal efficiency of each WWTP. In the final effluent of the N-WWTP we observed a large contribution of ibuprofen to the total amount of pharmaceuticals; this was not observed for the other WWTPs. 

### 3.2. Removal of Target Pharmaceuticals

As previously stated, pharmaceutical compounds have become a concerning group of emerging pollutants to the scientific community and their removal from wastewaters is essential to ensure the environmental quality of recipient ecosystems. Biologically-based WWTPs produce effluents that maintain water quality standards in order to reuse them or dispose into the environment at a reasonable cost, but these WWTPs have limited capability to remove pharmaceuticals [33]. For this reason, it is essential to evaluate the removal efficiency of different technologies, even more so if the purified wastewaters will be re-used in agriculture. To calculate the removal of the different treatments, the following equation was used in the different samplings and WWTPs surveyed.

$$RE\;\left(\%\right)=100-\left(\frac{\left[Effl\right]}{\left[Inf\right]}*100\right)$$

where *RE* is removal efficiency, [*Effl*] is the measured concentration of the pharmaceutical in the effluent of the treatment, and [*Inf*] is the measured concentration of the pharmaceutical in the influent of the treatment.

#### 3.2.1. Conventional Treatment WWTPs

In C-WWTP1, the secondary process provides median removals over 98% for the compounds with the highest concentrations (nicotine, caffeine, paraxanthine, ibuprofen, and naproxen). For the pharmaceuticals used in cardiovascular diseases, namely atenolol and gemfibrozil, the removal efficiency of the biological treatment was also great, with median removals over 72.5%. Diclofenac and trimethoprim showed slightly lower removal efficiencies of 42.9% and 66.5%, respectively. Only carbamazepine showed a negative value of removal. This means that the concentrations after the biological treatment were higher than in the influent. The observed persistence of carbamazepine in treated wastewaters has been the topic of many studies around the world, and for this reason, some authors like Hai et al. have proposed its use as an anthropogenic marker in water [34]. Other authors have attributed the poor removal of carbamazepine to its molecular structure and hydrophilicity [35,36,37]. In this WWTP, tertiary processes were also evaluated. The microfiltration process was not effective; in fact, the median removals were between –52.9% and 19.1%. Nevertheless, reverse osmosis was an effective method to remove these compounds. The removals of pharmaceuticals in this treatment were high, in all cases over 55%, even for persistent compounds such as carbamazepine. By comparing the concentrations of pharmaceuticals in influent and final effluent, the combination of biological treatment and tertiary processes based on reverse osmosis was effective in the removal of pharmaceutical residues. The median recoveries of the overall purification process were over 90% for all compounds under study, except carbamazepine, which showed an overall removal of 73.7%. This removal could be considered as very satisfactory in comparison with other studies in which the removal of this compound only reached 10–30% [38].

In C-WWTP2, another tertiary process was evaluated; in this case, the combination of an ultrafiltration and electrodialysis reversal process. There is very little literature about the efficiency of this treatment process in wastewaters. In our study, the monitoring was conducted for one year and the results showed that this technology has a moderate efficiency. Only four compounds (atenolol, naproxen, ibuprofen, and diclofenac) showed a median removal between 48% and 58%. The electrodialysis technology was not efficient with the rest of the compounds under study. The target stimulants, nicotine, caffeine, and paraxanthine, showed removal efficiencies between 13.8% and 25.5% while the efficiencies for trimethoprim, carbamazepine, and gemfibrozil were 20.4%, 12.3%, and 5.4%, respectively. These results complement the study of Arola et al. which established that using electrodialysis, diclofenac and ibuprofen were preferentially retained in the diluent [22]. However, the study of Arola et al. was done in a pilot plant and the authors established that the study must be confirmed using real wastewaters, like in this study.

#### 3.2.2. Natural Treatment WWTP

In this WWTP, the Imhoff process showed limited removal efficiency for pharmaceutical residues. In this sense, only two compounds showed 100% elimination during this process, but these two compounds, erythromycin and diclofenac, were detected in less than 20% of the surveyed samples; thus, it is not possible to ensure that this type of purification system is appropriate for the elimination of these two pharmaceuticals. Regarding the anti-inflammatories (naproxen and ibuprofen) and gemfibrozil, they showed negative elimination ratios, which means that the concentrations after the Imhoff process were higher than before. These negative elimination ratios could be explained by daily fluctuations in the concentrations of these compounds because the samples were taken at the same time in each sampling point. Furthermore, some deconjugation processes, in which conjugated compounds are converted into free compounds during the purification process, could explain the negative removal ratios obtained [7,39]. For the rest of the compounds, the eliminations were not high, and the median removals were between 2.9% and 41.8%. Regarding the constructed wetland processes, in most cases both configurations (vertical and horizontal flows) showed similar behaviors. In the vertical flow system, removals over 60% were achieved for the three stimulants, nicotine, caffeine and paraxanthine. Atenolol showed a medium removal of 51.4% and poor elimination efficiencies (between 10.6% and 33.1%) were obtained for trimethoprim, and the three anti-inflammatory compounds. Carbamazepine showed a trend similar to conventional secondary processes and the elimination was negative, which means that the concentration after the purification process was higher, as observed in other studies. In this vertical flow process, the concentrations of gemfibrozil were also higher after treatment which was also observed in a previous study on this WWTP [19]. Finally, the horizontal flow treatment provided slightly higher removal efficiencies than vertical flow treatment. In this sense, five compounds presented median eliminations over 75% (nicotine, caffeine, paraxanthine, atenolol, and naproxen). For trimethoprim and ibuprofen, the removals were low (30.0% and 26.4%, respectively), but higher than those obtained in the vertical flow systems. For diclofenac, a different trend was observed, because the median removal for this compound was –162.5%, which indicates an increase in the concentrations after this treatment which could be explained by the daily fluctuations or by deconjugation processes as in previous treatments. Finally, carbamazepine and gemfibrozil showed negative removals too, but the increase of the concentrations after the treatment was lower than in the vertical flow treatment. Overall, the natural treatment processes performed in this WWTP showed great eliminations for stimulants (over 97.5%), atenolol (90.9%), naproxen (79.4%), and trimethoprim (64.0%). Three compounds (carbamazepine, diclofenac, and gemfibrozil) showed negative removals after the whole purification system and this trend coincided with a previous study performed at this WWTP [19].

#### 3.2.3. Comparison between Conventional and Natural Purification Treatments

**Figure 3:**
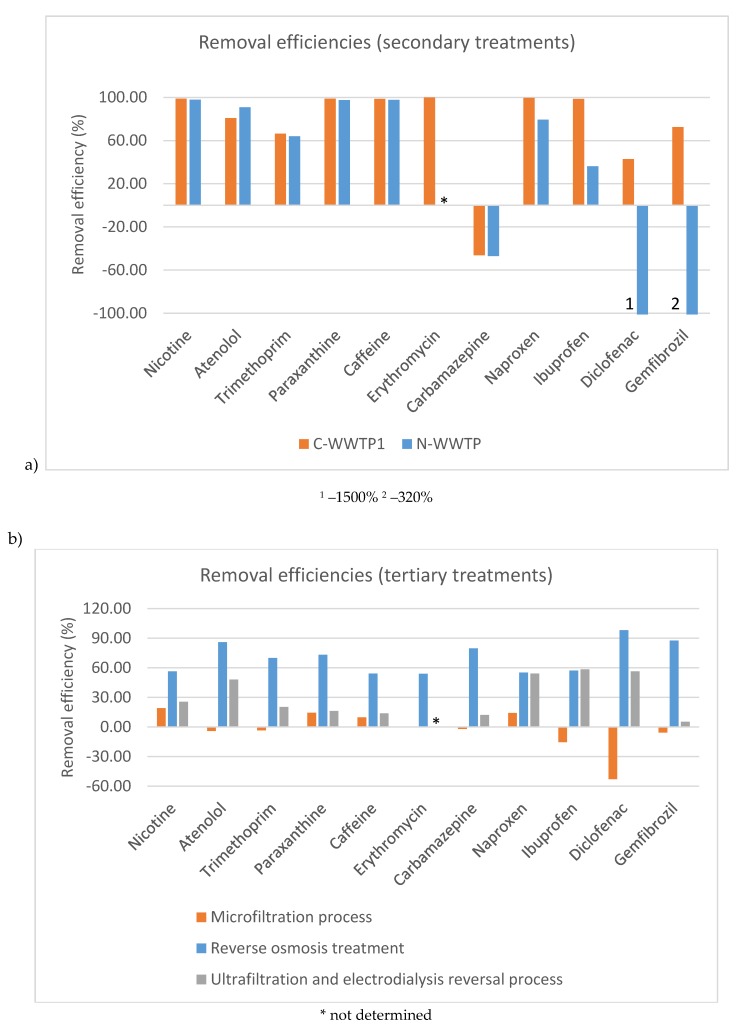
Median removal efficiencies for target compounds using (**a**) secondary treatments and (**b**) tertiary treatments.

To perform comparisons of the treatment technologies, between the two systems of conventional and natural purification, it is necessary to compare the same stages of purification. As can be seen in Figure 3a, both natural and conventional systems provide similar removal efficiencies for target compounds after secondary treatment. The highest elimination rates in both WWTPs were obtained for stimulants, with median removals over 97% and atenolol, which showed a median removal over 90% in both systems. Regarding anti-inflammatory compounds, the trends in the two surveyed WWTPs were different. Conventional treatments showed large removals for naproxen and ibuprofen (between 98.7% and 99.5%), while in the natural WWTP, the removals for these compounds were between 36.1% and 79.4%. For the third anti-inflammatory compound, diclofenac, C-WWTP1 showed a positive elimination ratio (42.9%) while in the N-WWTP, this efficiency was negative, which implies greater concentration of the free compound in the effluent of the system. In this sense, effluent concentrations of diclofenac were 10 times higher than influent concentrations, but in all cases below 0.5 µg·L^−1^. This trend was the same for gemfibrozil which showed a median elimination of 72.5% in C-WWTP1 and more than –300% in the N-WWTP. Finally, for trimethoprim, very similar median removals were achieved (in both wastewater treatment facilities it was over 60%) and this trend was also seen for carbamazepine which reflected the recalcitrant behavior of this compound with median removals near –50% in both WWTPs.

Regarding tertiary processes, it can be seen in Figure 3b that the reverse osmosis process was the most efficient purification process. The stimulants showed good removals for reverse osmosis process and not-satisfactory removals for microfiltration and electrodialysis reversal technologies. Regarding anti-inflammatories, similar removal efficiencies were obtained for naproxen and ibuprofen using reverse osmosis and electrodialysis reversal (between 54.1% and 58.3%) and for this family of compounds, it was stated that microfiltration technology was not appropriate for the elimination of them. For the rest of the compounds under study, in all cases the removal efficiencies were significantly better using reverse osmosis while electrodialysis reversal showed a better elimination capacity than microfiltration, but poor efficiency (below 30%).

## 4. Conclusions

In order to provide a safe and valuable resource of water in arid and semi-arid locations by using reclaimed water in agriculture, it is necessary to ensure its quality. One of the reluctances of farmers and legislators is related to the presence of some emerging pollutants, such as pharmaceutical residues, in treated waters. Although the concentration of these compounds in such waters are usually at trace levels, a deep knowledge about their presence and elimination is needed. 

Due to the singular characteristics of each family of target pharmaceutical compounds, their removal rates in the studied conventional purification treatments were very variable, but great efficiencies (up to 99.8%) were achieved for some of them. Satisfactory removal values were also provided by the studied natural treatment system, with comparable results for stimulants such as nicotine or caffeine and other drugs like atenolol and naproxen. However, for other compounds, namely ibuprofen, diclofenac, and gemfibrozil, natural treatments were not so effective as conventional ones. In addition, remarkable differences were also observed among tertiary technologies. Reverse osmosis revealed itself as a great option for the elimination of emerging pollutants. In this sense, the combination of secondary treatments and reverse osmosis provided very satisfactory removal efficiencies, over 95% for most compounds under study. Regarding other tertiary technologies, electrodialysis reversal also showed moderate removals for some pharmaceuticals, but in all cases, they were significantly lower than reverse osmosis.

Since all the target pharmaceutical compounds are still present in the studied treated water after using both conventional and natural systems (with concentration levels from ng·L^−1^ to µg·L^−1^), further studies are demanded in order to improve the purification systems. Special interest must be paid to some recalcitrant compounds, like carbamazepine, for which very low removal rates were achieved. 

The inclusion of emerging compounds, such as pharmaceuticals, in national and European contexts, is also mandatory in order to carry out adequate monitoring programs and to establish reliable control of these pollutants.

## Figures and Tables

**Table 1:** Names, identification numbers, molecular weights, and structures of target pharmaceuticals.

Drug Family	Compound	CAS No.	Molecular Weight (g/mole)	Structure
Stimulants	Nicotine	54-11-5	162.230	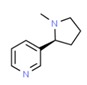
	Caffeine	58-08-2	194.191	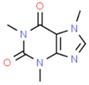
	Paraxanthine	611-59-6	180.164	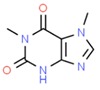
Anti-inflammatories	Naproxen	22204-53-1	230.260	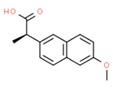
	Ibuprofen	15687-27-1	206.281	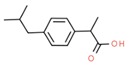
	Diclofenac	15307-86-5	296.149	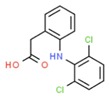
Lipid regulators	Gemfibrozil	25812-30-0	250.333	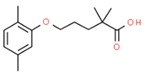
Anti-hypertensives	Atenolol	29122-68-7	266.336	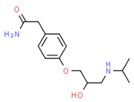
Anti-convulsants	Carbamazepine	298-46-4	236.269	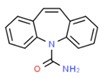
Antibiotics	Trimethoprim	738-70-5	290.318	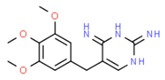
	Erythromycin	114-07-8	733.927	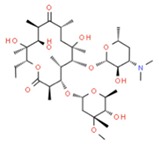

**Table 2:** Median concentrations, range of detected concentrations, and positive analysis for target pharmaceuticals. (**a**) C-WWTP1, (**b**) C-WWTP2, (**c**) N-WWTP.

(a)								
**Compound**	**Point A1**		**Point A2**		**Point A3**		**Point A4**	
	**Median Concentration (min–max Concentration)** **(µg·L^−1^)**	**Positive Results (%)**	**Median Concentration (min–max Concentration)** **(ng·L^−1^)**	**Positive Results (%)**	**Median Concentration (min–max Concentration)** **(ng·L^−1^)**	**Positive Results (%)**	**Median Concentration (min–max Concentration)** **(ng·L^−1^)**	**Positive Results (%)**
Nicotine	14.179 (0.005–45.82)	89.5	0.151 (0.064–0.449)	88.9	0.136 (0.002–0.586)	86.7	0.065 (0.001–0.251)	87.5
Atenolol	1.134 (0.354–2.899)	84.2	0.218 (0.006–0.643)	83.3	0.287 (0.007–1.949)	86.7	0.061 (0.001–0.088)	81.3
Trimethoprim	0.231 (0.053–0.658)	84.2	0.077 (0.034–0.157)	77.8	0.071 (0.029–0.169)	73.3	0.021 (0.013–0.054)	75.0
Paraxanthine	47.869 (12.46–95.63)	89.5	0.518 (0.101–6.900)	88.9	0.425 (0.036–5.901)	86.7	0.117 (0.031–1.262)	81.3
Caffeine	36.495 (15.47–72.62)	89.5	0.589 (0.095–2.499)	88.9	0.663 (0.120–2.705)	80.0	0.169 (0.051–1.943)	87.5
Erythromycin	0.083 (0.070–0.094)	15.8	0.067 (0.043–0.090)	11.1	0.052 (0.044–0.709)	26.7	0.047 (0.047–0.073)	25.0
Carbamazepine	0.146 (0.045–2.394)	89.5	0.240 (0.137–2.597)	88.9	0.234 (0.078–4.687)	86.7	0.052 (0.013–0.158)	87.5
Naproxen	27.333 (4.910–521.7)	84.2	0.172 (0.072–1.528)	66.7	0.282 (0.057–1.311)	73.3	0.102 (0.011–0.584)	68.8
Ibuprofen	19.894 (0.128–147.5)	84.2	0.208 (0.047–1.585)	77.8	0.214 (0.009–1.184)	80.0	0.139 (0.005–1.751)	56.3
Diclofenac	0.139 (0.006–0.708)	47.4	0.117 (0.007–0.404)	77.8	0.152 (0.034–0.288)	73.3	0.024 (0.006–0.115)	37.5
Gemfibrozil	3.353 (0.053–40.63)	84.2	0.729 (0.012–3.602)	83.3	0.598 (0.033–2.865)	80.0	0.103 (0.004–0.485)	56.3
(b)								
	**Point B1**		**Point B2**					
**Compound**	**Median Concentration (min–max Concentration)** **(µg·L^−1^)**	**Positive Results (%)**	**Median Concentration (min–max Concentration)** **(ng·L^−1^)**	**Positive Results (%)**				
Nicotine	0.103 (0.038–0.228)	100	0.105 (0.047–0.343)	100				
Atenolol	0.286 (0.001–0.521)	100	0.184 (0.167–0.234)	85.7				
Trimethoprim	0.055 (0.014–0.130)	100	0.043 (0.014–0.074)	100				
Paraxanthine	0.310 (0.037–1.256)	100	0.241 (0.031–2.634)	100				
Caffeine	0.378 (0.058–0.817)	100	0.369 (0.050–2.749)	100				
Erythromycin	nd *	0.0	nd *	0.0				
Carbamazepine	0.209 (0.148–0.312)	85.7	0.193 (0.107–0.327)	85.7				
Naproxen	0.198 (0.150–0.237)	57.1	0.116 (0.077–0.555)	57.1				
Ibuprofen	0.125 (0.027–0.173)	100	0.074 (0.008–0.969)	85.7				
Diclofenac	0.202 (0.070–0.280)	85.7	0.091 (0.027–0.139)	85.7				
Gemfibrozil	0.723 (0.392–1.098)	85.7	0.701 (0.306–0.883)	85.7				
(c)								
	**Point C1**		**Point C2**		**Point C3**		**Point C4**	
**Compound**	**Median Concentration (min–max Concentration)** **(µg·L^−1^)**	**Positive Results (%)**	**Median Concentration (min–max Concentration)** **(ng·L^−1^)**	**Positive Results (%)**	**Median Concentration (min–max Concentration)** **(ng·L^−1^)**	**Positive Results (%)**	**Median Concentration (min–max Concentration)** **(ng·L^−1^)**	**Positive Results (%)**
Nicotine	9.606 (6.449–63.94)	89.5	7.651 (0.194–37.29)	89.5	3.515 (0.134–12.70)	88.9	0.245 (0.086–1.077)	81.8
Atenolol	2.195 (0.757–10.00)	84.2	1.402 (0.037–2.396)	89.5	0.742 (0.072–1.990)	83.3	0.198 (0.082–0.753)	81.8
Trimethoprim	0.034 (0.017–2.166)	63.2	0.031 (0.013–0.531)	68.4	0.020 (0.014–0.674)	50.0	0.018 (0.015–0.088)	36.4
Paraxanthine	40.050 (9.619–85.77)	89.5	21.243 (6.867–39.84)	89.5	5.084 (0.143–30.56)	88.9	0.596 (0.151–2.480)	81.8
Caffeine	37.888 (10.39–126.4)	89.5	41.497 (11.16–65.22)	89.5	16.771 (0.382–47.58)	88.9	1.633 (0.261–5.595)	81.8
Erythromycin	0.101 (0.071–0.131)	10.5	0.066 (0.025–0.107)	10.5	0.081 (0.045–5.346)	33.3	0.064 (0.063–0.066)	18.2
Carbamazepine	0.299 (0.039–1.306)	89.5	0.306 (0.081–0.969)	89.5	0.432 (0.069–7.797)	88.9	0.604 (0.247–1.009)	81.8
Naproxen	9.653 (0.911–320.1)	84.2	9.383 (2.581–177.0)	84.2	7.713 (0.644–77.47)	83.3	1.397 (0.310–5.179)	81.8
Ibuprofen	16.942 (4.378–121.4)	84.2	21.886 (5.331–133.7)	84.2	14.209 (0.931–43.97)	83.3	9.803 (6.658–18.76)	81.8
Diclofenac	0.035 (0.008–0.055)	21.1	0.028 (0.007–0.718)	47.4	0.139 (0.024–0.591)	61.1	0.525 (0.020–2.813)	81.8
Gemfibrozil	0.222 (0.001–16.11)	73.7	0.647 (0.006–5.632)	78.9	2.904 (0.112–7.403)	83.3	3.972 (1.104–7.670)	81.8

* nd: not detected.

## Supplementary Materials

The following are available online, Table S1: Gradient used for the chromatographic separation of target pharmaceuticals, title, Table S2: Parent and fragmentation ions and collision conditions for the mass spectrometry detection of target pharmaceuticals.
