# Supplementary material for: A Survey of the Presence of Pharmaceutical Residues in Wastewaters. Evaluation of Their Removal using Conventional and Natural Treatment Procedures
Source: Molecules. 2020 Apr 2;25(7):1639. doi: 10.3390/molecules25071639 (PMC7180812; doi:10.3390/molecules25071639)
Supplement: Supplementary file 1 [file molecules-25-01639-s001.pdf]

Table S1. Gradient used for the chromatographic separation of target pharmaceuticals

Time (min)	Flow ($mL \cdot min^{-1}$)	% A (Water + 0.5% acetic acid)	% B (Methanol + 0.5% acetic acid)
0.00	0.300	90	10
0.56	0.300	90	10
3.83	0.300	40	60
6.93	0.300	10	90
7.42	0.300	10	90
7.91	0.300	90	10
9.00	0.300	90	10

Table S2. Parent and fragmentation ions and collision conditions for the mass spectrometry detection of target pharmaceuticals.

Compound	Ionization mode	Cone voltage (V)	Parent ion	Quantification ion (collision energy, V)	Confirmation ion (collision energy, V)
Nicotine	ESI+	25	163.1	130.1 (20)	117.1 (20)
Atenolol	ESI+	35	267.2	145.1 (25)	190.1 (15)
Trimethoprim	ESI+	40	291.3	123.1 (25)	230.3 (20)
Paraxanthine	ESI+	30	181.1	124.1 (20)	96.0 (20)
Caffeine	ESI+	35	195.1	138.1 (20)	110.1 (20)
Erythromycin	ESI+	35	734.5	158.2 (30)	576.5 (20)
Carbamazepine	ESI+	35	237.2	194.2 (20)	192.2 (20)
Naproxen	ESI–	10	229.2	170.2 (20)	185.2 (10)
Ibuprofen	ESI–	20	205.2	161.2 (10)	-
Diclofenac	ESI–	20	294.2	250.2 (10)	214.2 (20)
Gemfibrozil	ESI–	20	249.2	121.1 (20)	-
Atenolol D7	ESI+	30	274.4	145.1 (30)	79.1 (20)
Sulfamethoxazole D4	ESI–	20	256.2	160.1 (20)	96.1 (30)
Ibuprofen D3	ESI–	20	208.2	164.2 (10)	79.8 (20)
